# Modelling of longitudinally cut carrot curling induced by the vascular cylinder-cortex interference pressure

**DOI:** 10.1098/rsos.230420

**Published:** 2024-01-24

**Authors:** Nguyen A. Vo-Bui, Benedict A. Rogers, Elise C. Pegg

**Affiliations:** ^1^ Department of Mechanical Engineering, University of Bath, Claverton Down, Bath BA2 7AY, UK; ^2^ ART_AI CDT, Department of Computer Science, University of Bath, Claverton Down, Bath BA2 7AY, UK

**Keywords:** carrots, bending, curl, residual stress, root pressure

## Abstract

Cut carrot pieces are popular convenience foods, which enable the use of misshapen or physiologically imperfect produce. Cut carrots curl due to residual stress, which limits their shelf life and causes unnecessary food waste. The aim of this study is to identify the geometrical and environmental factors which have the most influence on their longevity. An analytical equation was developed using compound cylinder solutions, and this was used to define carrot-specific finite element (FE) models. Over 100 longitudinally cut Lancashire Nantes carrot halves were characterized, each was modelled analytically and verified using FE models. This model was evaluated by comparing predicted curvatures to ones experimentally measured over a week. The average radius of curvature decreased from 1.61 to 1.1 m a week after. A 1.32× reduction in the elastic modulus after 7 days was observed. The moisture content reduction relates to 22% weight loss, correlating to the decreasing radius of curvature. Subsequently, carrots are recommended to be stored in humidity-controlled environments. The experimental results from this study match the predictions made using mechanical principles. The research provides a methodology to predict the deformation of cut root vegetables, and the procedure is likely to be applicable to other plant structures.

## 1. Introduction

*Daucus carota* L., henceforth carrot, is one of the highest market value crops in the world and is noted for its high production efficiency [1,2]. Despite this, carrot wastage is high. Approximately 25–30% of this occurs prior to processing and packaging due to physiological deformities, mechanical damage or infected sections [3]. Fresh cut and minimally processed carrots are sold as a convenient ready-to-use ingredient, and these products also have the advantage of making use of carrots which might otherwise have been discarded, thus reducing food waste. However, minimally processed carrots are highly sensitive to storage conditions, such as humidity and temperature, because they influence residual stresses within the root vegetable, as well as affecting the microbiological environment [4]. Carrots, like many other vegetables, are also susceptible to mechanical damage during packaging and transport [5,6]. For these reasons identifying the optimal packaging for fresh cut carrots presents a significant challenge. The curl of cut carrots can be a cause for disposal of such products and the deformation may result from poor packaging, handling or improper storage conditions. We propose that the curl of cut carrots can be represented using standard mechanical engineering principles to provide simple tools to help food manufacturers predict and mitigate against carrot wastage from curling. This study uses a combination of analytical approaches (compound cylinder calculations) and numerical methods (finite element (FE) analysis) to trial this approach and tests the predictions against gathered experimental data. The nomenclature used throughout this paper is outlined in table 1.

**Table 1.** Nomenclature.

CAD	computer-aided design			
FE	finite element			
A	area		[m^2^]	
E	elastic modulus		[Pa]	
M	moment		[Nm]	
p	pressure		[Pa]	
R	radius of curvature		[m]	
r	radius of stem		[m]	
X	calibration factor		[dimensionless]	
y	displacement		[m]	
*ε*	strain		[dimensionless]	
*θ*	angle		[rad]	
*μ*	moisture content		[%]	
*σ*	stress		[Pa]	

## 2. Background

In order to apply mechanical engineering principles to carrots, it is important to know the properties of carrot as a material in mechanical terms. Numerous studies have quantified the material properties of carrots using mechanical testing. For instance, Jahanbakhshi *et al.* [7] measured the mean geometric diameter, density and the minimum force to bruise carrots. Xia *et al*. [8] also studied the bruise behaviour of carrot under impact loading. Thiel & Donald [9] performed tensile testing and reported the longitudinal elastic modulus for fresh carrots to be 1.36 MPa. After one week, this value decays to 1.03 MPa. Stopa *et al.* [10,11] conducted a lateral load test and measured strain using pattern interferometry, which gives a more accurate measure. They concluded that the elastic moduli for carrots was nearly four times higher than that measured by Thiel & Donald [9]. In this study, we decided to base our material model on data from Stopa *et al.* [10]. However, it is worth noting that both the approaches these studies used [9,10] to measure deformation resulting from residual stresses are invasive and focus on single points to infer the stress state of the whole sample. Such an approach requires the assumption of distributions between measurement points, and we are keen to avoid this assumption in our experimental study. To ensure an accurate model is obtained, a visual measurement method was selected for this study, which gives a full-length impression of the curvature.

FE modelling is an established tool in mechanical and civil engineering and is gaining increasing popularity in the agriculture sector [12,13] because it allows projection of interior stress states that would not be accessible with physical tests. FE modelling has been used to study the mechanical loading thresholds which cause bruising in carrots [10] and has also been used to identify areas prone to mechanical damage post harvesting [11], yet this has not been used to model the curling of carrots. In this study, we test the hypothesis that the curling of carrots is purely mechanical and can be represented as such using FE models.

**Figure 1.**
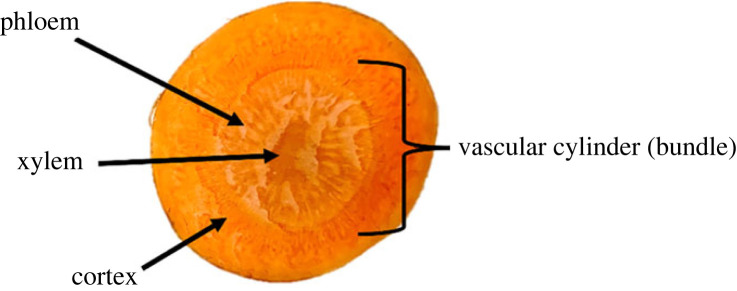
Carrot anatomy.

To do this, we have modelled the carrot using a cylindrical coordinate system and with orthotropic material properties. Modelling of woody plants as an orthotropic material commonly uses nine constants, including the elastic moduli in the hoop, radial and longitudinal directions and those relating to Poisson's ratios and shear behaviour [14]. Many studies modelled wood treating the vascular cylinder and the cortex as separate layers [10,11,15,16]. Lamé's equation for cylinders was often incorporated in the multi-layered model to compute residual stress resulting from diametral interference between layers [15]. As such, it was considered reasonable to model carrots in terms of the vascular cylinder, the transport system of the root vegetable which consists of xylem and phloem; and the cortex, the surrounding layer where nutrients are stored (figure 1).

It was observed in the work of Santos *et al*. [17] that the vascular bundle of a carrot undergoes far less shrinkage during drying than the cortex. This will cause an imbalance of contraction in carrots when cut lengthways, inducing a bending moment. For that reason, it was anticipated that carrots would curl with a smaller radius as time passed and moisture escaped.

There have been studies on the stress states resulting from the interference pressure between layers in carrots [18,19]. However, how this stress state deforms the root vegetable after cutting has yet to be investigated, hence the original contribution of this paper. This study proposes a model to characterize the root pressure caused by the misfit of layers, and how this pressure drives the curling behaviour. To investigate this behaviour, the following objectives were determined:

— to catalogue data on the progression of carrot curling after longitudinal cutting,
— to propose a model of the curls observed caused by the residual stresses within the root layers, using Euler's theory of beam bending and Lamé's compound cylinder solutions, and
— to use the FE method to verify the proposed theory against visual data.

A numerical approach has been proposed to use a single curvature radius model to describe the curling behaviour. The key benefit of this approach is that it will allow the estimation of the interface pressure without introducing excessive variables or noise, which will give a simple explanatory model for the behaviour and allow mitigation strategies to be inferred. A validated FE representation of the curling behaviour of carrots will enable growers and other food processing business to improve packaging and storage, and thus reduce food waste [8,10,11,13].

## 3. Methods

The study involved measuring deformation patterns during and after a carrot-cutting experiment to investigate how the carrots curl when chopped lengthwise. These data were used in conjunction with a FE model to validate the proposed theory. A notation CXXHX will be used throughout this work, where the former three letters refer to the carrot number and the latter two refer to the half number (e.g. C10H2 indicates carrot number 10, half number 2).

### 3.1. Radius of curvature measurement

Fifty-two fresh Nantes carrots (Brookfield Farm, Lancashire, UK) of varying sizes were stored in zip lock bags in a refrigerator (AUCL 4884W) at 3°C. Each carrot was labelled and marked with a pen at positions 10 mm and 20 mm from the crown end and then in intervals of 20 mm until the tip end. The diameters at the marked position were measured with a vernier calliper with an accuracy of 0.02 mm. The length and mass of each carrot was also recorded using a ruler to the nearest mm and a weighing scale (OPKS009, One Home Collection) of resolution 1 g. The mass recorded was used to measure the evaporable moisture content *µ*, in a method analogous to the approach taken by Jahanbakhshi *et al.* [7] and Shahgholi *et al*. [13], with

3.1 $$\mu =\frac{m_{\mathrm{initial}}-m_{\mathrm{instantaneous}}}{m_{\mathrm{initial}}}\;\times 100\text{\%},$$

where $m_{\mathrm{initial}}$ is the initial mass of the carrot half, $m_{\mathrm{instantaneous}}$ is the dried mass at time *t*.

Each carrot was chopped in half with a sharp chef's knife. Each carrot half was clamped lightly on a lab stand such that the cut plane was levelled with the 18 megapixel camera (Coolpix S9400, Nikon, fitted with a Nikkor 18× wide optical zoom lens VR set to 25 mm focus (or zero-zoom)). The set-up was levelled using a spirit level on the cut surface at the crown tip. One image was taken for each carrot half before the carrot half was stored in a zip lock bag in the refrigerator. The bags counteract the dried refrigerator's natural convection, which prolongs the lifespan of the carrots by lowering the root vegetables' hydration [20]. The halves were placed along the vertical edge of the bags to ensure the curling action of carrots can occur as naturally as possible. The procedure was repeated for all carrot halves on the 3^rd^ and 7^th^ day after the initial cut.

**Figure 2.**
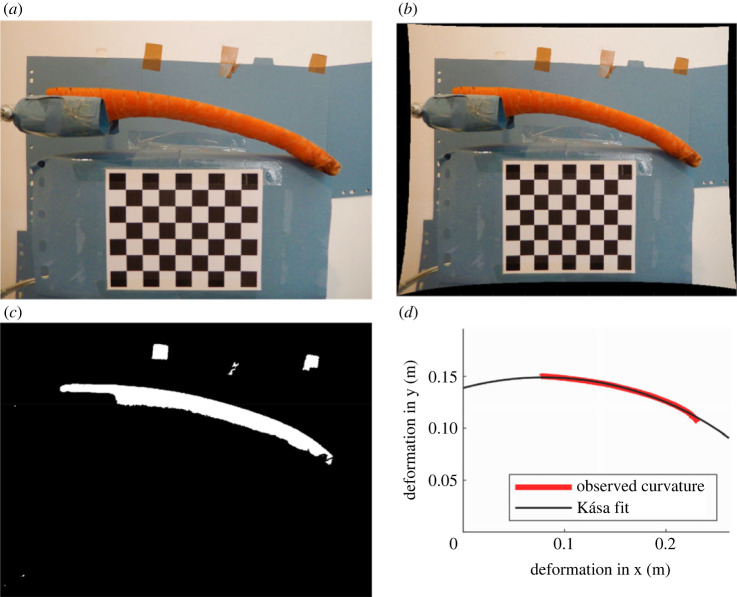
Image processing workflow: (*a*) original image; (*b*) chequerboard calibrated image; (*c*) binary image; (*d*) radius of curvature, estimated using Kása fit [21] on the upper edge of the curl.

The measurement workspace made use of a blue background to maximize the contrast of carrots when applying a thresholding algorithm to the images (figure 2*a*). As camera lenses often suffer from curvilinear distortion due to their shape, Zhang's method of camera calibration [22] was used to ensure real straight lines remain straight on the image. A printed chequerboard pattern of 63 squares of size 10 mm was printed on card held in line with the plane of the carrot centreline in the images to allow removal of this distortion (figure 2*b*).

**Figure 3.**
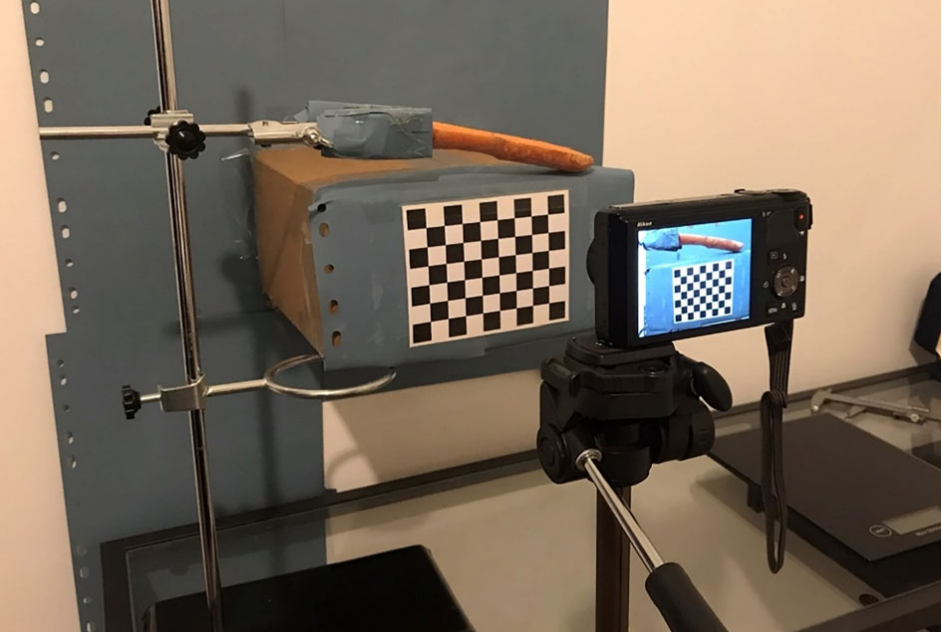
The apparatus used for measuring the carrot curvatures.

To segment the carrot half from the image background, Otsu's method [23] was used to generate binary images of each carrot (figure 2*c*). A Matlab script was written to extract from the image the upper edge of a carrot half as a position vector. From this the radius of curvature could be estimated using the second-order Kása circle fitting procedure [21], as depicted in figure 2*d*. Figure 3 shows the overall measurement set-up.

### 3.2. Core diameter measurement

Carrots in this study were modelled as two regions: a vascular cylinder and a cortex, as both are important in determining the root vegetable's bulk stiffness [10,11]. The diameter of the vascular cylinder could not be measured accurately from the longitudinally cut carrots because the cut plane was not always perfectly in the centre, so a separate experiment was performed using cross-sectional cuts.

In determining the relative core size, nine carrots were marked at 10 and 20 mm from the crown, then at intervals of 20 mm along the centreline. At the marked positions, the carrots were chopped crosswise, and the vascular cylinder diameters were recorded with the same vernier calliper (0.02 mm resolution). These data were used to correlate the core-to-outer diameter ratios to the relative distance along the carrot length in order to define the model geometries.

### 3.3. Modelling methods

To estimate the interference load caused by carrot growth, an expression for the pressure at the interface of the cortex and the vascular cylinder was derived from first principles (§3.3.1). The root pressure distribution along the carrot was then implemented in the FE model as a boundary load condition (§3.3.2).

#### 3.3.1. Finding the root pressure

A single curvature radius approach was proposed to model the carrot halves, which correlates the root pressure to the bending radius through the use of classical beam-bending theory alongside Lamé's solution to residual stress arising from interference of the cortex-vascular bulk.

**Figure 4.**
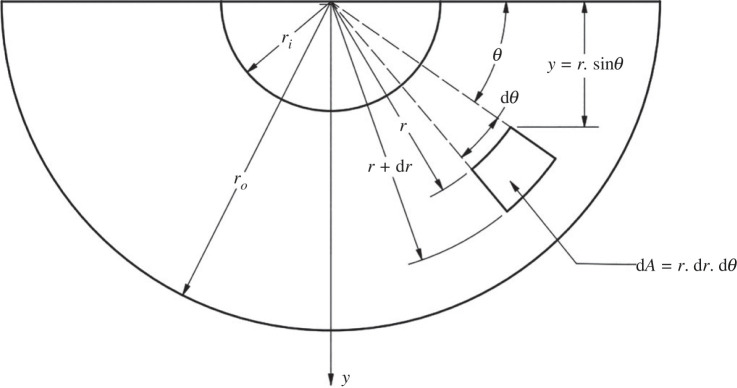
An infinitesimal element in a transverse section.

Consider an infinitesimal element in pure bending (figure 4). The bending moment acting on the element can be calculated by equation (3.2), where $M_{c}$ is the total bending moment acting on an area *A* that caused the carrot half to bend, $\sigma_{c}$ is the bending stress at distance *y* from the neutral axis, $E_{c}$ is the longitudinal elastic modulus of the cortex, ε is the axial direct strain, *r* and *θ* are the radial and angular positions, *R* is the radius of curvature.

3.2 $$dM_{c}=y\sigma_{c}dA=yE_{c}\varepsilon dA={\left(r\sin\theta \right)}^{2}\frac{E_{c}}{R}r.dr.d\theta .$$

Expansion of equation (3.2) gives

3.3 $$dM_{c}=\frac{E_{c}}{R}r^{3}\sin^{2}\theta .dr.d\theta .$$

By isolating the vascular bulk from the cortex and integrating both sides with the condition zero area has zero moments, the bending moments experienced by the cortex from interference of the cortex-vascular bulk can be calculated as

3.4 $$M_{c}=\int_{0}^{\pi }\int_{r_{i}}^{r_{o}}\frac{E_{c}}{R}r^{3}\sin^{2}\theta .dr.d\theta =\frac{\pi E_{c}}{8R}(r_{o}^{4}-r_{i}^{4}).$$

This bending moment could be linked to work out the root pressure $p_{i}$ inside the carrots through Lamé's solution for thick cylinders. The hoop and radial stresses, $\sigma_{\theta }$ and $\sigma_{r}$, in the cylindrical coordinate system are expressed in equations (3.5) and (3.6), respectively.

3.5 $$\sigma_{\theta }=p_{i}\left[\frac{r_{i}^{2}}{r_{o}^{2}-r_{i}^{2}}\left(1+\frac{r_{o}^{2}}{r^{2}}\right)\right]$$

and

3.6 $$\sigma_{r}=p_{i}\left[\frac{r_{i}^{2}}{r_{o}^{2}-r_{i}^{2}}\left(1-\frac{r_{o}^{2}}{r^{2}}\right)\right].$$

Taking moments across the longitudinal axis of the carrots,

3.7 $$M=\sigma .y.dA=\int_{0}^{\pi }\int_{r_{i}}^{r_{o}}(\sigma_{\theta }+\sigma_{r}).r.\sin\theta .dr.d\theta .$$

Substituting the Lamé's solutions (equations (3.5) and (3.6)) into equation (3.7) gives

3.8 $$M=4\int_{r_{i}}^{r_{o}}\;p_{i}.\frac{r_{i}^{2}}{r_{o}^{2}-r_{i}^{2}}.r.dr=2p_{i}r_{i}^{2}.$$

This moment *M* caused by carrot root pressure is assumed to be directly proportional to the observed moment $M_{c}$ causing the carrot to bend by

3.9 $$M_{c}=X\times M=2Xp_{i}r_{i}^{2},$$

where *X* is the curling factor to account for inner shear effects within the carrot halves caused by the vascular bundle. Linking equations (3.4), (3.8) and (3.9), the root pressure could be evaluated from the measured radius of curvature as

3.10 $$p_{i}=\frac{\pi E_{c}(r_{o}^{4}-r_{i}^{4})}{16Xr_{i}^{2}R}.$$

The local root pressures were computed from equation (3.10) as boundary conditions for the FE models.

#### 3.3.2. Finite element analysis

Three-dimensional computer-aided design (CAD) models of the carrots were made according to the geometric data. These models were made based on the assumptions that carrots' central axes were perfectly straight, and the carrots were axisymmetric. Any irregularities such as lateral scars were assumed to be negligible. The model assumed that the carrot halves are split longitudinally perfectly through the centre. The CAD models were then imported into Ansys Mechanical v. 2020 R2 for structural analysis. The models were discretized using Ansys hexahedral quadratic element SOLID186, which is recognized to have better deformation accuracy in beams than its linear or tetrahedral counterparts [24]. A mesh of size 1.5 mm was chosen after performing a mesh convergence study.

**Figure 5.**
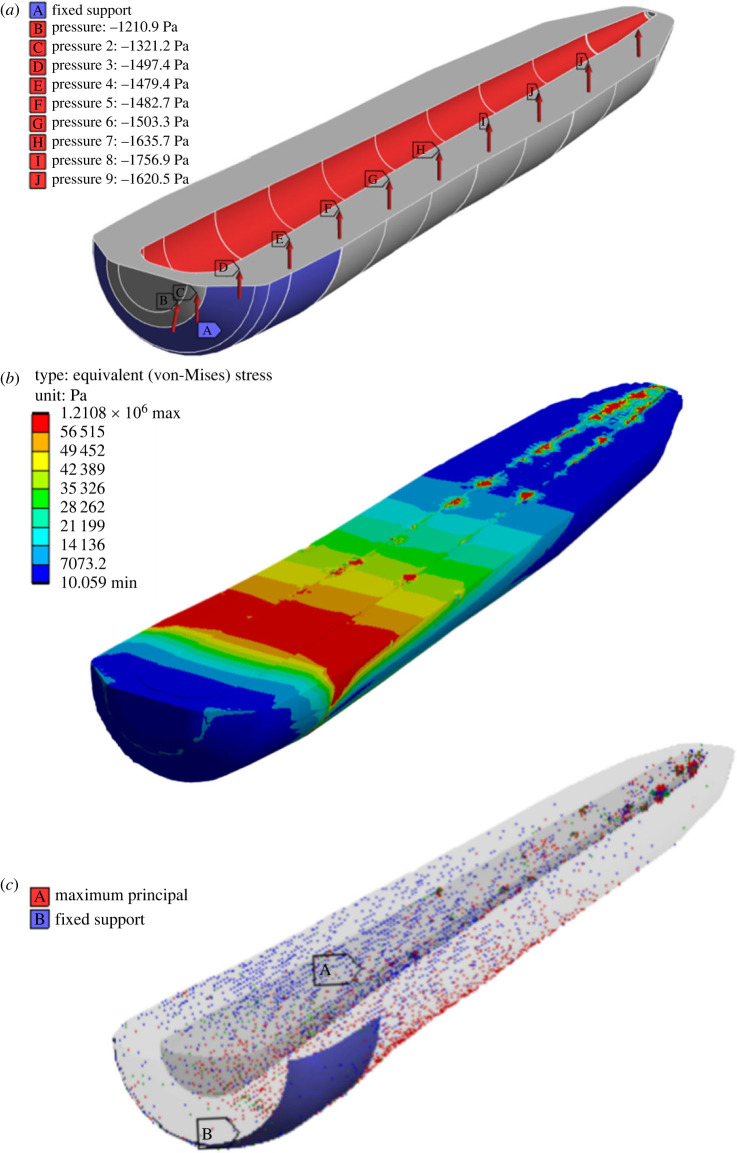
The FE workflow: (*a*) root pressure distribution is applied to the cortex; (*b*) stress distribution is evaluated; (*c*) the residual stress distribution is mapped on a released carrot model.

Using equation (3.10) with an arbitrary *X* factor, a root pressure distribution could be obtained. This distribution was then applied on the inner surfaces of the carrot's cortex (see electronic supplementary material, appendix 1). Fixed boundary conditions were applied on the outer surfaces axially between 10 and 20 mm from the crown (figure 5*a*). Upon solving the initial problem, the stress state (figure 5*b*) was mapped onto a similar carrot model and stress released was observed (figure 5*c*).

The carrot's mechanical properties used in this model were taken from Stopa *et al.* [10] with the drop in elastic moduli within 7 days [9] interpolated, as outlined in table 2.

**Table 2.** Simulated material properties.

day	component	$E_{xx}$(MPa)	$E_{yy}$(MPa)	$E_{zz}$ (MPa)	$\nu_{xy}$	$\nu_{yz}$	$\nu_{zx}$	$G_{xy}$(MPa)	$G_{yz}$ (MPa)	$G_{zx}$ (MPa)
1	vascular bundle [10]	4.76	4.62	4.88	0.473	0.482	0.468	1.62	1.56	1.66
	cortex [10]	4.49	4.33	4.85	0.475	0.475	0.477	1.52	1.47	1.64
3	vascular bundle	4.38	4.25	4.49	0.473	0.482	0.468	1.49	1.43	1.53
	cortex	4.13	3.98	4.46	0.475	0.475	0.477	1.4	1.35	1.51
7	vascular bundle	3.61	3.50	3.70	0.473	0.482	0.468	1.22	1.18	1.26
	cortex	3.40	3.28	3.67	0.475	0.475	0.477	1.15	1.11	1.24

**Figure 6.**
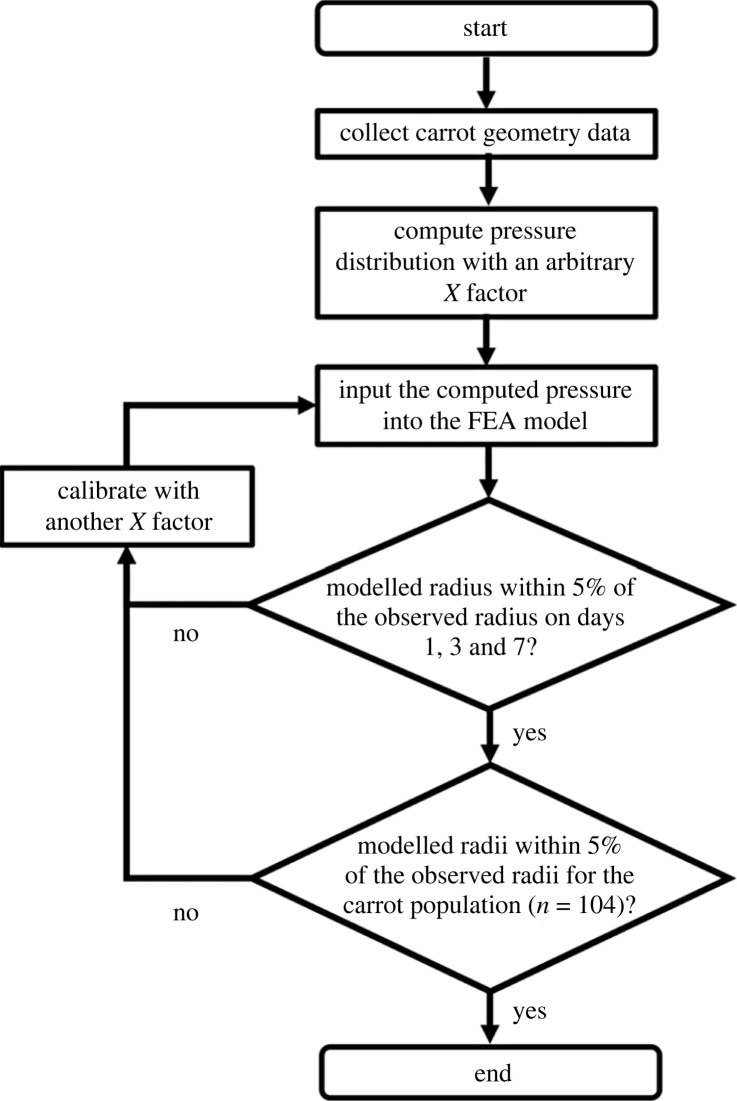
The single curvature radius workflow.

The FE method outlined was used to solve the models iteratively on a subset of carrot geometries to calibrate for the *X* factor, which resulted in a simulated bending radii within 5% of the observed radii. The calibrated *X* factor was then applied to the remaining 104 carrot geometries to validate the model. The overall workflow of the model is outlined in figure 6.

## 4. Results and discussion

### 4.1. Radius of curvature

**Figure 7.**
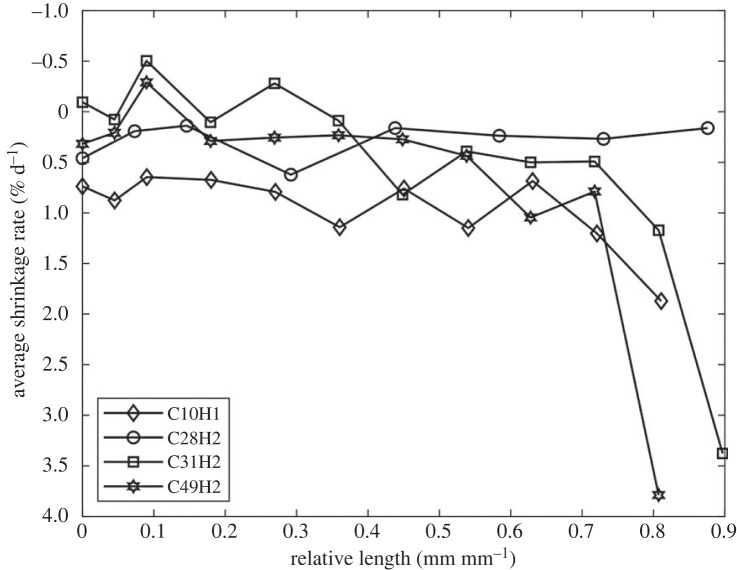
Carrot shrinkage rate against relative length (average across 7 days).

**Figure 8.**
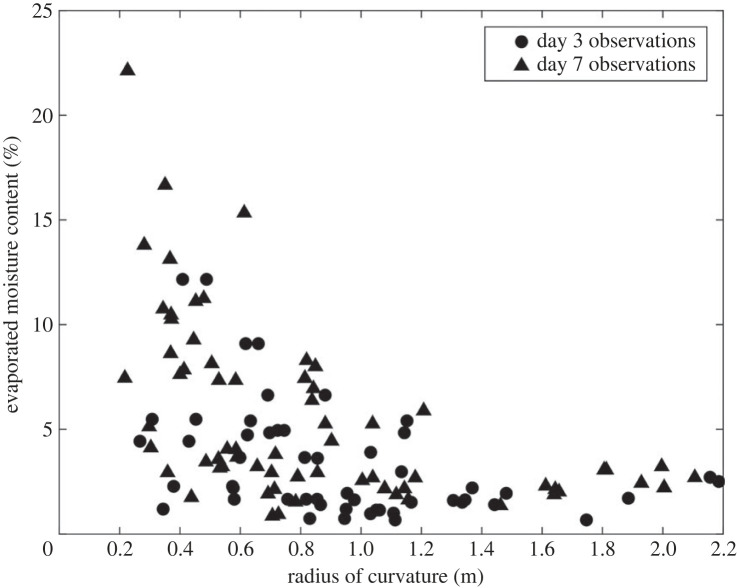
Evaporated moisture content versus radius of curvature on days 3 and 7.

The average radius of curvature decreases from 1.61 m a day after cutting, to 1.38 m after 3 days and 1.14 m after a week. Further, the carrots' diameters were observed to change throughout the monitoring period of 7 days. The mean diametral shrinkage rate fluctuated around 0.37% per day. Carrot lengths were all normalized against the average carrot length (termed ‘relative length’) to enable comparison. A proportion of carrots were observed to shrink significantly at the narrow tip (for instance C10H1, C31H2 and C49H2, figure 7). Furthermore, as the carrot halves dehydrated, they were observed to bend more (figure 8).

### 4.2. Core diameter estimation

**Figure 9.**
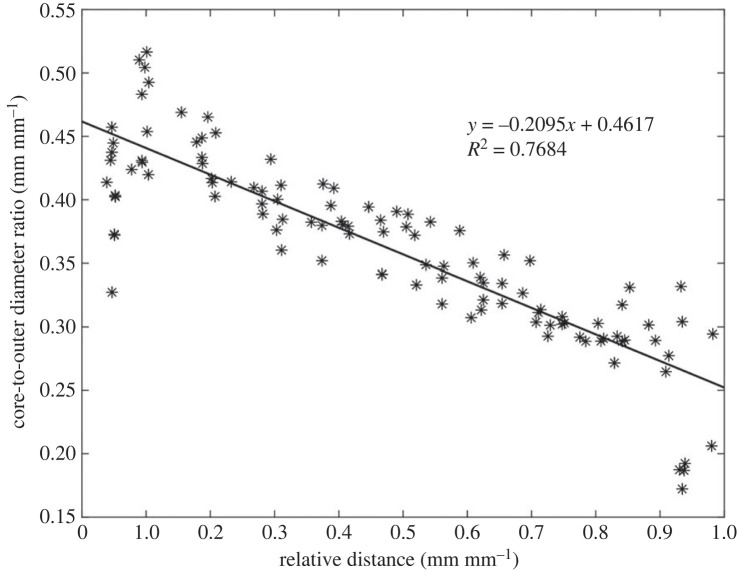
Core-to-outer diameter carrot ratio against normalized carrot length.

Figure 9 illustrates the core-to-outer diameter ratio with carrot lengths normalized. The vascular cylinder to cortex outer diameter ratio decreases linearly from the plant end towards the narrow tip of the carrot. The *R*^2^ value of 0.77 suggests that there is a negative correlation between the diameter ratio to the relative length of the carrot. A linear model was used to describe the core size of the carrot population for modelling purposes.

### 4.3. Model results and verification

**Figure 10.**
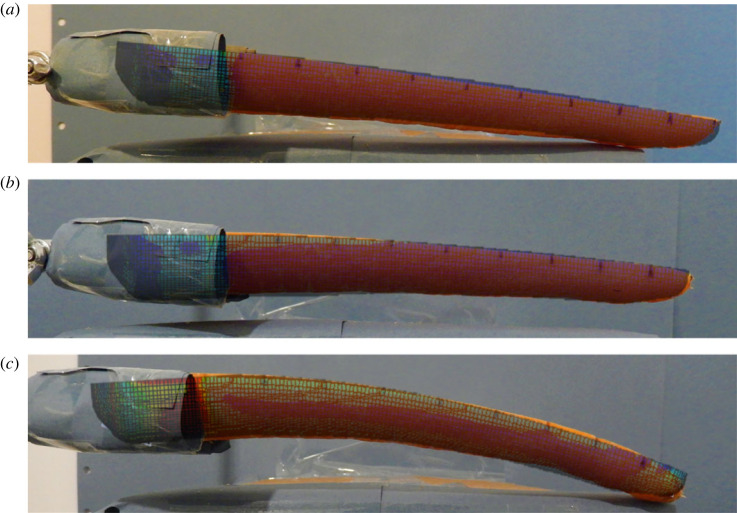
Photographs of sample C31H2 on days 1 (*a*), 3 (*b*) and 7 (*c*) superimposed with their respective FE results to visually illustrate how the single-curvature model can predict the curl of cut carrots.

With the *X* factor of 0.5, the FE models were enforced such that the computed deformation match with that of measured in Experiment 1 on days 1, 3 and 7. Figure 10 shows the FE results superimposed the physical testing images for C31H2.

**Figure 11.**
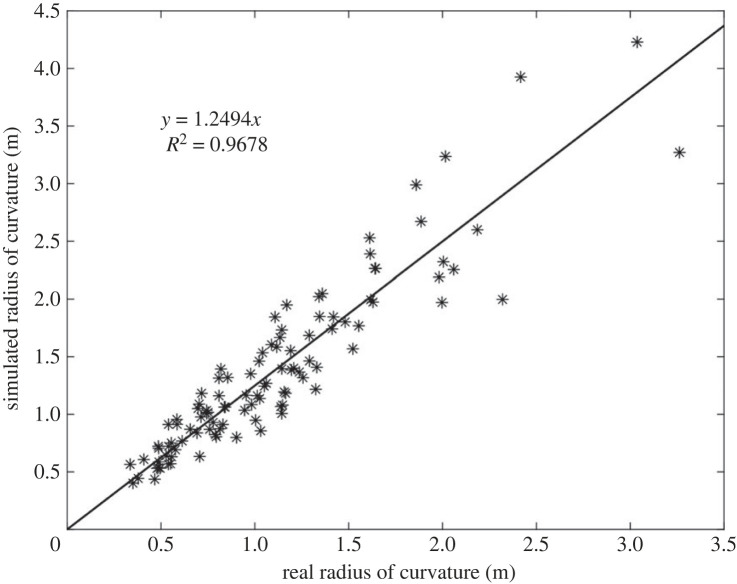
Measured radius of curvature against simulated radius.

The *X* factor of 0.5 was verified by looping the deformation results for all 104 carrot halves (see supplementary data). There is a strong linear correlation (*R*^2^ = 0.97) between the observed radii and the simulated radii (figure 11), suggesting that the theory proposed can reliably predict the curl of a cut carrot and that the deformation is driven by the initial root pressure. Note that the gradient of the line of best fit should be 1, and the gradient of 1.25 suggests that there is a slight overestimation bias in our calibration factor.

**Figure 12.**
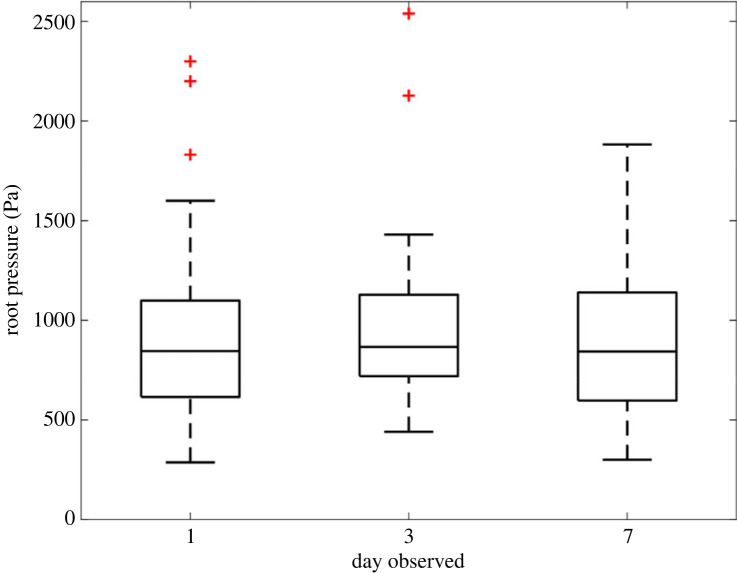
Box plot of root pressure (whole carrot population) against time.

The model suggests that the root pressure encounters negligible changes with time (figure 12) when the decrease in elastic moduli over time observed by Thiel & Donald [9] is taken into account.

**Figure 13.**
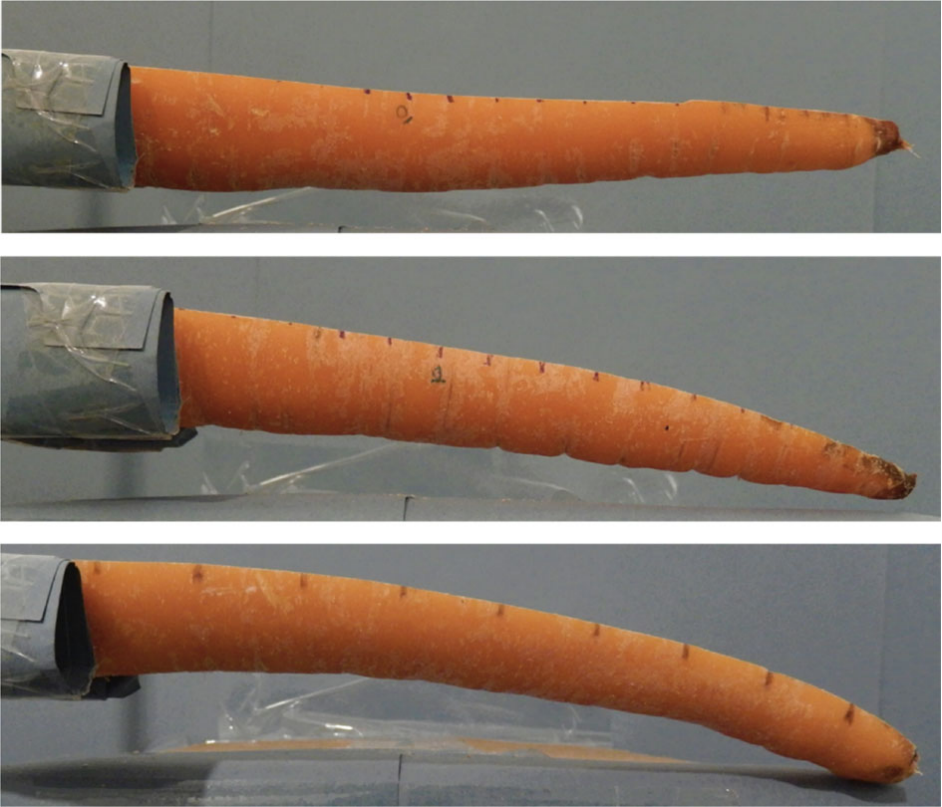
Tip browning seen on some halves.

In general, the proposed model in this paper works well with the carrot population. The hypothesis derived from Santos *et al*. [17] of increased curvature with time was found to hold up well in the data. With a calibration factor of 0.5 the model was able to represent the vegetable population. Using one calibration factor allowed the FE models to conform to the image data, which further validates the drop in Young's modulus concluded by Thiel & Donald [9]. The models had some systematic error which caused a slight increase in the radius of curvature of the FE models. This may arise from the treatment of the carrot tip. Often the high surface area to volume ratio of the tips could be seen to cause them to dehydrate and contract (figure 13), affecting the curvature caused by the internal stresses. This effect was not reflected in the FE model and is expected to be the primary cause for carrots being stiffer in the model than in measurements. Other factors which could lead to differences between the FE models and the experiment data include:

— The pressure distributions were generated based on the longitudinal elastic modulus $E_{c}$ with an assumption of isotropy in material properties (equation (3.11)), whereas the materials used in FE are orthotropic, and some level of anisotropy can be expected [10,11].
— The pressure distribution was computed by assuming the vascular cylinders have negligible tensile strength and have no capability of pulling or pushing the carrot halves.
— The shear effects and coupled Poisson's effect are ignored in the classical beam bending theory.

A fundamental assumption made by the beam model was that the use of a single curvature radius would be a sufficient model to estimate the interface pressure. This was initially made as a judgement call with the aim of finding a model which could explain the curling behaviour without the need for complex empirical variables, or introducing datasets to the point of capturing noise instead of usable trends. While the assumption did lead to around 11.8% of measurement events being inexplicable, it can be seen in figures 9 and 11 to have been a suitable assumption to allow conclusions to be drawn on the causes of the behaviour. Despite the limitations of the model, the correlation between the experimental and model data was high (*R*^2^ = 0.97) and the systematic error could be addressed by including an offset into the calculations.

The model proposed was able to replicate the real curvatures of the carrot halves; however, there were some outliers in the analysis which were observed to be stiffer than the actual carrots. These outliers were often observed to have their tips decolourized (figure 13), most likely from reacting to oxygen inside the refrigerator [25]. This resulted from the carrots being long enough that the seals on the zip lock bags were not effective. As a consequence, the elastic moduli at the tip might have been reduced from that expected of the carrot at the given time [9]. Those halves with their tips browned were usually from the longer carrots, particularly those with lengths greater than 206 mm, and this was shown to be significant (see electronic supplementary material, appendix 2). This greatly increased the curvature on a local level and reinforces the importance of the storage conditions to maintain carrot properties for the maximum amount of time.

The predicted interference pressure at the cortex–vascular cylinder interface correlated well with the trend observed in the literature. The dropped pressure from day 7 matched with how Kokkoras [18] described the drying effect of carrots. As they dry, the decrease in water means that there is lower incompressible water content within the cells and in the inter-cell space, which leads to a decrease in turgor pressure within the cell wall and consequently causes a decrease in the root interface pressure. It is encouraging that the model is able to replicate known biological effects and this indicates that the curling of the carrots is being driven by a combination of mechanical and biological factors.

**Figure 14.**
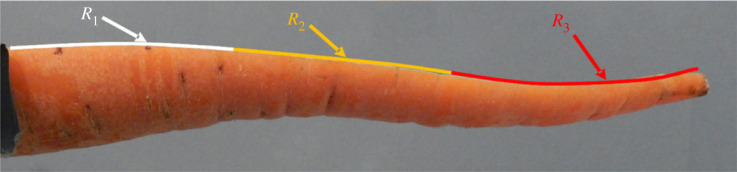
An example of complex bending behaviour in carrots.

There are many inherent limitations and assumptions in the analyses performed in this study. The model proposed in this work was based on a fundamental assumption: that the bending of carrot halves form a circular arc. This is not always the case, as seen in figure 14. Of the specimens tested, there were halves that bent in a complex manner which did not allow an accurate estimation of the root pressure. For these curls, the pressure could be computed by segmenting the carrot cut profile. Deformation could then be estimated locally in the smaller sections (figure 14). From this, it would be possible to find the local root pressures and the bending shape could be more accurately represented in the FE model. This length split argument could be extrapolated to smaller segments similar to the mesh size and investigated for further causal factors of bending behaviour. Despite the promise of the method in theory, this approach was not pursued because in the experiments 88.2% of the assessed samples displayed simple bending. This allowed a simplified explainable model based on the circular arc to be assumed for analysis. In creating the theorized compound model, many variables would need to be introduced. It was judged likely that the curvature measurements would as such capture local noise to fully adhere to the complex shape, which would severely hamper efforts to find a causal explanation for the curvature phenomenon.

Another ideal assumption involved in this proposed model is that carrots are assumed to be cut symmetrically about the main axis and that the cutting process does not interfere with the initial stress state of the carrots. This is difficult to achieve especially when carrots naturally do not grow symmetrically, their roots are likely to be grown in different shapes or forms. In addition, the scope of these experiments was limited to Nantes carrots, the classical beam bending and compound cylinder theory may not hold for other carrot varieties such as the Paris Market or the Chantenay species where the length to diameter aspect ratio is much lower than the ones assessed in the described experiments. For such carrots with low aspect ratios, the Timoshenko–Ehrenfest beam theory, which takes into account the shear deformation and rotational bending effects, could be more suitable.

While the scope of this study was limited to carrots, mechanical engineering principles have been applied to other produce [6,26] which indicates the approach could be expanded to cover many vegetable structures. Further, the model could be applied to wider biological layered structures such as timber [15] and protruding stems (e.g. bamboo), subject to experimental validation. These materials hold crucial load bearing roles and the ability to mechanically explain and predict bending when bisected would be of benefit to construction and design.

## 5. Conclusion

This study has provided a mechanical explanation for the bending of longitudinally cut carrots using Lamé's model of concentric cylinders. Carrot halves were observed to bend along their axis when chopped lengthways, and the bending model detailed in this paper was able to replicate the radius of curvature of deformed carrots measured experimentally with a strong linear correlation (*R*^2^ = 0.97). The decay factor of 1.32 in elastic moduli observed experimentally within the investigation period was also verified by the FE model, and trends in the predicted interface pressure between the cortex and the vascular cylinder correlated well with botanical literature. There were some outlier samples for which the FE models overestimated the stiffness especially at the narrow tips. These were found to be samples which had been oxidized, and this had accelerated the decay in stiffness. Compound bending behaviour was also apparent in a minority of samples and the circular bending model did not properly capture bending behaviour in these carrot halves. It is intended to address this using a sectional model and explore a wider range of geometries using Timoshenko–Ehrenfest beam bending theory in a future work. This study has, however, shown the main mechanisms behind the curl of cut carrots and detailed a method by which the phenomenon can be predicted numerically. This study allows us to recommend that manufacturers, in order to preserve carrots' mechanical properties, should handle carrots in cold, moist and airtight environments. These models can be used to help design packaging and food handling processes, and so reduce food waste.

## Data Availability

The dataset produced from this research can be found at https://doi.org/10.15125/BATH-01340 [27]. Included in this package are the full dataset of carrot and stress measurements, the images used to measure the curls of the carrot population, the images from the FE model and the Matlab code used for analysis. Supplementary material is available online [28].
